# Prioritizing solutions to incorporate Prosthetics and Orthotics services into Iranian health benefits package: Using an analytic hierarchy process

**DOI:** 10.1371/journal.pone.0253001

**Published:** 2021-06-08

**Authors:** Saeed Shahabi, Shahina Pardhan, Ahmad Ahmadi Teymourlouy, Dimitrios Skempes, Shabnam Shahali, Parviz Mojgani, Maryam Jalali, Kamran Bagheri Lankarani

**Affiliations:** 1 Health Policy Research Center, Institute of Health, Shiraz University of Medical Sciences, Shiraz, Iran; 2 Department of Health Services Management, School of Health Management and Information Sciences, Iran University of Medical Sciences, Tehran, Iran; 3 Vision and Eye Research Unit (VERU), School of Medicine, Anglia Ruskin University, Chelmsford, United Kingdom; 4 Disability Policy and Implementation Research Group, Swiss Paraplegic Research (SPF), Nottwil, Switzerland; 5 Rehabilitation Research Center, Department of Physiotherapy, School of Rehabilitation Sciences, Iran University of Medical Sciences, Tehran, Iran; 6 Iran-Helal Institute of Applied Science and Technology, Tehran, Iran; 7 Research Center for Emergency and Disaster Resilience, Red Crescent Society of The Islamic Republic of Iran, Tehran, Iran; 8 Rehabilitation Research Center, Department of Orthotics and Prosthetics, School of Rehabilitation Sciences, Iran University of Medical Sciences, Tehran, Iran; University of Central Florida, UNITED STATES

## Abstract

**Introduction:**

Health benefits package (HBP) is regarded as one of the main dimensions of health financing strategy. Even with increasing demands for prosthetics and orthotics (P&O) services to approximately 0.5% of the world’s population, only 15% of vulnerable groups have the chance to make use of such benefits. Inadequate coverage of P&O services in the HBP is accordingly one of the leading reasons for this situation in many countries, including Iran.

**Aims:**

The main objective of this study was to find and prioritize solutions in order to facilitate and promote P&O services in the Iranian HBP.

**Study design:**

A mixed-methods (qualitative-quantitative) research design was employed in this study.

**Methods:**

This study was conducted in two phases. First, semi-structured interviews were undertaken to retrieve potential solutions. Then an analytic hierarchy process (AHP) reflecting on seven criteria of acceptability, effectiveness, time, cost, feasibility, burden of disease, and fairness was performed to prioritize them.

**Results:**

In total, 26 individuals participated in semi-structured interviews and several policy solutions were proposed. Following the AHP, preventive interventions, infant-specific interventions, inpatient interventions, interventions until 6 years of age, and emergency interventions gained the highest priority to incorporate in the Iranian HBP.

**Conclusion:**

A number of policy solutions were explored and prioritized for P&O services in the Iranian HBP. Our findings provide a framework for decision- and policy-makers in Iran and other countries aiming to curb the financial burdens of P&O users, especially in vulnerable groups.

## Introduction

Prostheses are utilized to replace one part or the whole limb deficiency, and orthoses aim to correct and support the structure and function of the musculoskeletal system [1, 2]. As part of health care services, prosthetics and orthotics (P&O) services are often assumed in the domain of rehabilitation, oriented towards enabling users to achieve maximum performance and autonomy [3, 4]. According to the World Health Organization (WHO) estimate, about 0.5% of the world’s population is in need of P&O services [5]. In addition, evidence suggests that the given rate will reach 1% in the near future [6].

Despite these needs, proper access to high-quality P&O services without any financial hardships is facing serious challenges in most parts of the world, even in high-income countries [6, 7]. Only up to 15% of in need groups have the chance to make use of such benefits [8], which can disrupt progress towards Universal Health Coverage (UHC) and Sustainable Development Goals (SDGs). In view of this situation, the WHO is striving to promote rehabilitation services including those of P&O in health care systems, through initiating several guidelines like the Global Disability Action Plan (GDAP) 2014–2021 [9] and Rehabilitation in Health Systems (RHS) [10] as well as Rehabilitation 2030: A Call for Action [11]. Furthermore, in accordance with the Convention on the Rights of Persons with Disabilities (CRPD), member states are obligated to secure the availability of assistive devices including prostheses and orthoses for target groups, especially individuals with disabilities [12].

In Iran, as a developing country facing substantial economic and social issues, P&O services are rarely covered by health insurance companies or only a small portion of the costs are considered in specific cases [13]. In addition, that small coverage is provided mainly through private and supplementary insurances, which are only available to high-income groups [14]. Given that many service users are from poor and disabled groups, the financial burdens are enormous. In response, the Iranian Parliament has recently enacted the Law on the Protection of the Rights of Persons with Disabilities, in which, according to Article 6 of this law, rehabilitation services must be covered by health insurance companies [15].

Various approaches are being used to control health costs and to increase equity on the way towards UHC, such as designing health benefits package (HBP) [16], as one of the main dimensions of health financing strategy [17]. HBP is considered as a set of services that can be feasibly financed and provided under the actual circumstances in which a given country finds itself [18]. Different countries have incorporated various interventions into the HBP [19]. Priority-setting in health can be defined as the task of determining the priority to be assigned to a service, a service development, or an individual patient at a given point in time [20]. In this field, there are two types of prioritization: health problems and heath solutions or interventions [21]. Whereas it is not possible to cover all services, prioritization as a prerequisite for inclusion of services into the HBP is of utmost importance [22]. This study aimed to find and prioritize potential solutions in order to facilitate and promote P&O services in the Iranian HBP using a mixed-methods research design. To the best of the authors’ knowledge, this study was the first one regarding P&O services.

## Material and methods

**Fig 1:**
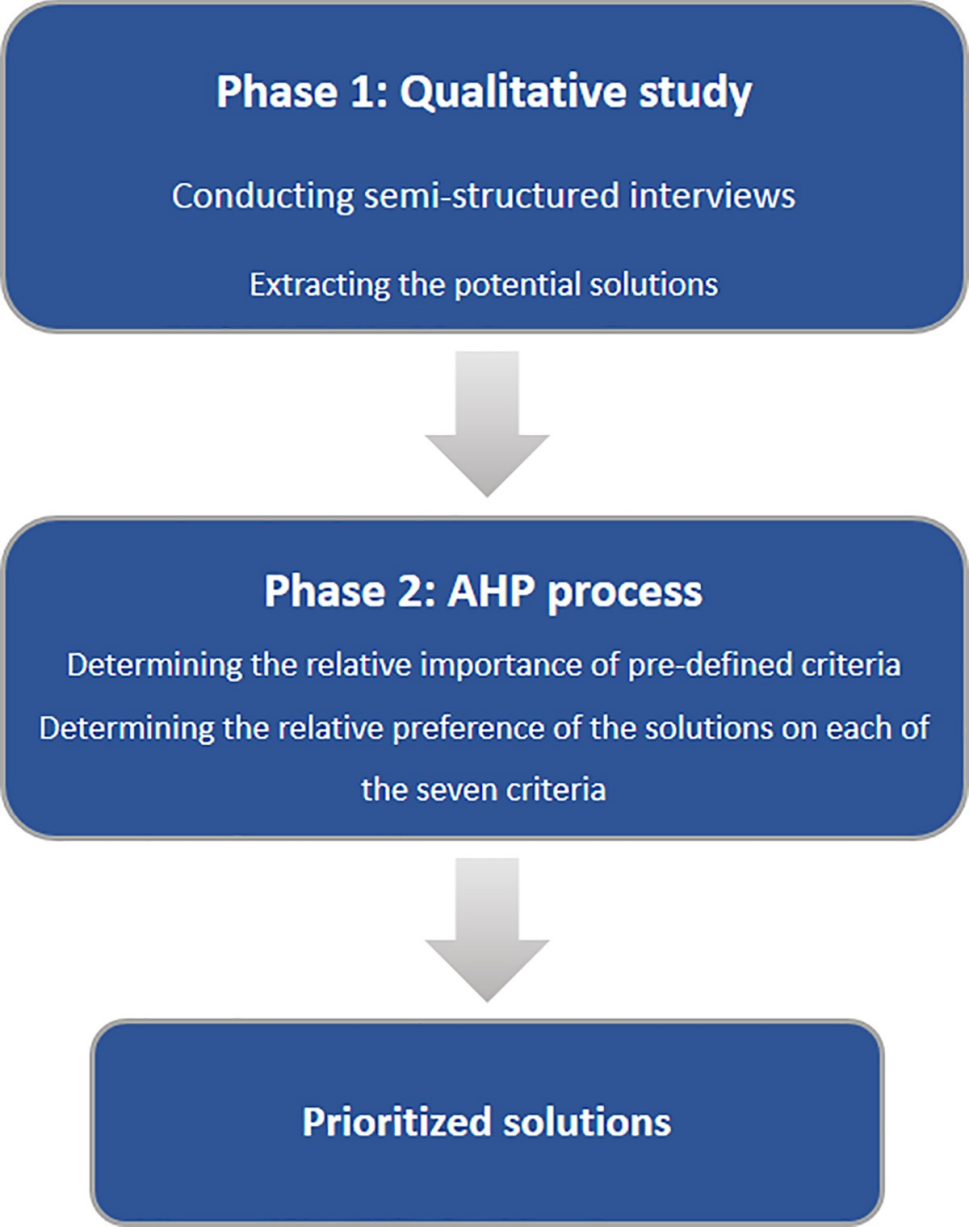
Overview of study methods.

This study was part of a larger project conducted using a mixed-methods research design at Iran University of Medical Sciences (IUMS). As illustrated in Fig 1, firstly, a qualitative study was completed to find potential solutions, and then an AHP was performed in order to prioritize them. The protocol of this study had been previously approved by the Institutional Review Board (IRB) of IUMS.

### Phase I: Qualitative study

Initially, semi-structured interviews were undertaken by the first author (viz. a male health policy-maker with a background in the field of rehabilitation) to identify and capture potential solutions to consider P&O services in the Iranian HBP. To this end, the research team sought to receive perspectives from Prosthetists/Orthotists, faculty members, and health policy-makers regarding solutions to incorporate P&O services into the HBP. These individuals were selected using purposive and snowball sampling methods with maximum variation in terms of specialty, geographic location, gender, professional experience, and employment status (namely, private or public).

Prosthetists and Orthotists practicing and having at least four years’ clinical experience were eligible criteria for participating. They should also have a related formal academic certification from the medical universities and a registration number of the Medical Council of the Islamic Republic of Iran (IRIMC). Furthermore, to receive in-depth descriptions, faculty members were recruited from five Orthotic and Prosthetic departments across the country. They should have at least three years’ experience in theoretical and clinical educations. In relation to health policy-makers, we tried to select individuals who are familiar with the health care system and the insurance mechanisms, as well as with physical rehabilitation sector. To set the interview schedule, the first author sent a written informed form and an invitation letter through an instant messaging application and e-mail. Also, the interviewer presented the information related to the study verbally at the beginning of each session. Face-to-face interviews were accordingly performed in a quiet room at the workplace of the participants living in the cities of Tehran, Shiraz, and Isfahan. Interviews with individuals residing in other regions were conducted via phone calls and the Internet. All interview sessions were organized using a guide consisting of open-ended questions and they were recorded digitally from January to June 2019. Sampling continued until data saturation was achieved, which was approved by evaluating the findings of the last two interviews to recognize duplicate data. After each session, the recorded files and notes were transcribed and saved anonymously in the Microsoft Office to facilitate the analysis process. The six-step procedure developed by Braun and Clarke was employed for thematic analysis [23]. To promote the rigor and trustworthiness of findings, various techniques such as member-checking by participants and co-authors, peer debriefing, data triangulation, and involvement of several authors in the analysis process were conducted [24].

### Phase II: AHP

**Fig 2:**
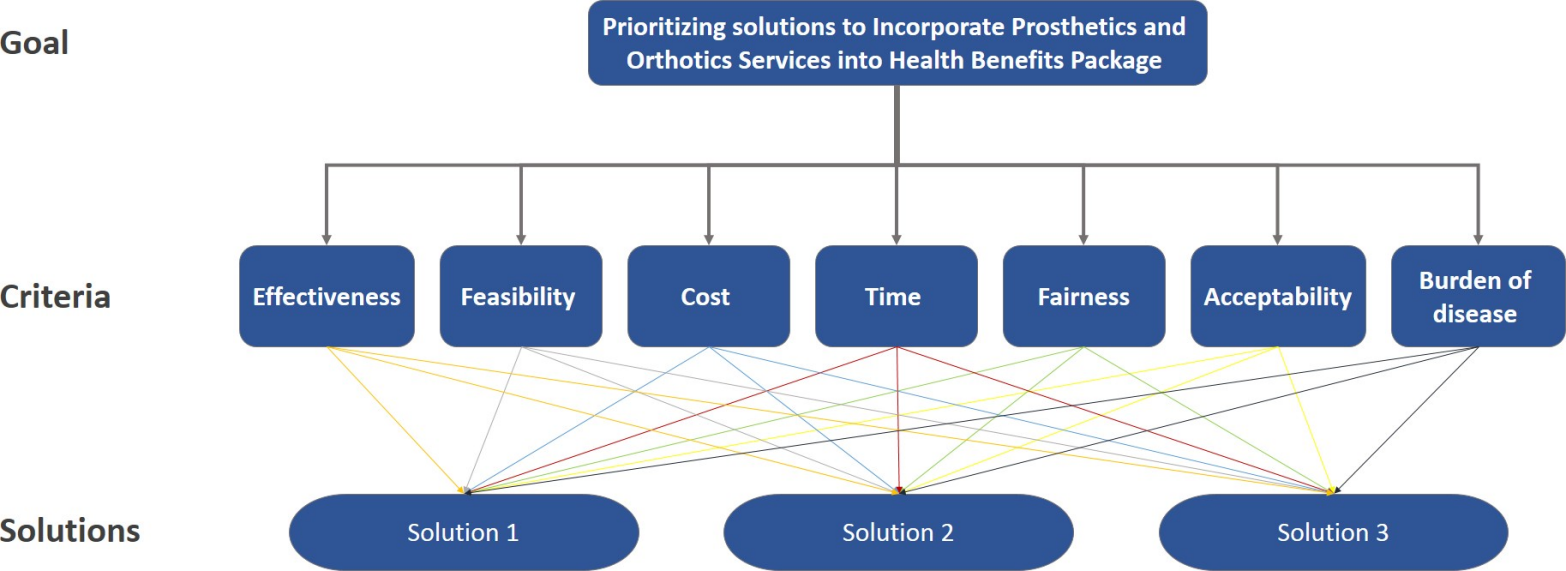
An analytic hierarchy process of final goal, desirable criteria, and potential solutions.

In the second phase, the AHP was used to prioritize the identified solutions. Indeed, this multi-criteria decision-making (MCDM) approach, applying pairwise comparisons to give weight to the desired criteria and comparing the proposed alternatives with the weighted criteria, aimed to assign the best solutions (Fig 2) [25]. In accordance with the WHO prioritization guideline, seven criteria including acceptability, effectiveness, time, cost, feasibility, burden of disease, and fairness were adopted [26]. To specify the relative importance of the aforementioned criteria, judgments by ten experts including health policy-makers, health insurers, faculty members, rehabilitation administrators, and practitioners were received by sending a matrix through e-mails by S.SH. Although evidence suggests that an expert’s viewpoint is sufficient in an AHP survey [27], depending on the study objectives, there were attempts to select various relevant experts with different scientific and healthcare backgrounds. A recommended nine-point ratio scale was further utilized to obtain the judgments [27, 28]. The levels of preference on this scale were as follows: 1 (equal importance), 3 (moderate importance), 5 (strong importance); 7 (very strong importance), and 9 (extreme importance). Additionally, 2, 4, 6, and 8 indicated intermediate values. The data were then aggregated and the geometric logarithmic mean was calculated for each pairwise comparison. These calculated values were employed to show the relative importance of the desired criteria and to recognize the weighted preferences for the solutions. Moreover, inconsistency of the participants’ responses, indicating how much the collected data could be trusted, was computed for each comparison. Based on evidence, an inconsistency index of ≤ 0.1 could be appropriate and acceptable [29]. The AHP was also fulfilled by S.SH, employing Expert Choice (version 11) software (Arlington, Virginia, USA).

## Ethical approval

Following the initial approval of Institutional Review Board (IRB) of School of Health Management and Information Sciences, this project was approved by the Ethical Committee of the Iran University of Medical Sciences, Tehran, Iran (IR-IUMS-REC-1397-889). All contributors gave a signed written informed consent form.

### Results

In total, 26 individuals including eight health policy-makers, six faculty members, and 13 Prosthetists/Orthotists agreed to participate in the semi-structured interviews (S1 Table), and 14 policy solutions were suggested. The retrieved solutions were then prioritized for being incorporated in the Iranian HBP using the AHP with regard to the seven recommended criteria of acceptability, effectiveness, time, cost, feasibility, burden of disease, and fairness (Table 1). Overall, preventive interventions, infant-specific interventions, inpatient interventions, and interventions until 6 years of age obtained the highest priority; respectively.

**Table 1:** Potential solutions and their weighted preferences to consider the P&O services in the Iranian HBP.

ID	Solutions	Rates							
		Acceptability	Effectiveness	Time	Cost	Feasibility	Burden of disease	Fairness	Overall
**1**	Cost-effective interventions	8	9	10	9	9	12	11	11
**2**	Preventive interventions	2	2	1	1	1	3	6	1
**3**	Golden period interventions	9	4	2	2	5	4	10	6
**4**	Long-term interventions	11	12	12	12	12	2	9	10
**5**	Inpatient interventions	1	5	4	5	2	1	2	3
**6**	Crucial prosthetic interventions	6	8	9	10	10	6	1	8
**7**	Infant-specific interventions	3	1	5	3	3	5	5	2
**8**	Interventions until 6 years old	5	6	6	4	4	8	3	4
**9**	Interventions until 18 years old	7	7	7	6	7	10	8	7
**10**	Aging-specific interventions	10	10	8	8	8	9	7	9
**11**	Cosmetic prostheses	14	13	13	13	14	13	13	14
**12**	High-tech interventions	13	14	14	14	13	14	14	13
**13**	Sport interventions	12	11	11	11	11	11	12	12
**14**	Emergency interventions	4	3	3	7	6	7	4	5

In accordance with the pairwise comparison of the selected criteria regarding the final goal, the burden of disease with a ratio of 0.259, fairness with a ratio of 0.215, and effectiveness with a ratio of 0.212 had the highest relative importance; respectively. These criteria were followed by feasibility with a ratio of 0.126, cost with a ratio of 0.084, time with a ratio of 0.056, and acceptability with a ratio of 0.048 (Table 2). The inconsistency index of this pairwise comparison was 0.05. Then, paired comparison of each solution was conducted according to the selected criteria.

**Table 2:** Matrix of pairwise comparisons.

Main criterion	Acceptability	Effectiveness	Time	Cost	Feasibility	Burden of disease	Fairness	Relative importance
**Acceptability**	1	1/4	1/2	1/2	1/2	1/5	1/3	0.048
**Effectiveness**	4	1	3	3	3	1	1/2	0.212
**Time**	2	1/3	1	1/3	1/3	1/5	1/3	0.056
**Cost**	2	1/3	3	1	1/2	1/4	1/3	0.084
**Feasibility**	2	1/3	3	2	1	1/3	1	0.126
**Burden of disease**	5	1	5	4	3	1	1	0.259
**Fairness**	3	2	3	3	1	1	1	0.215

### Acceptability

**Fig 3:**
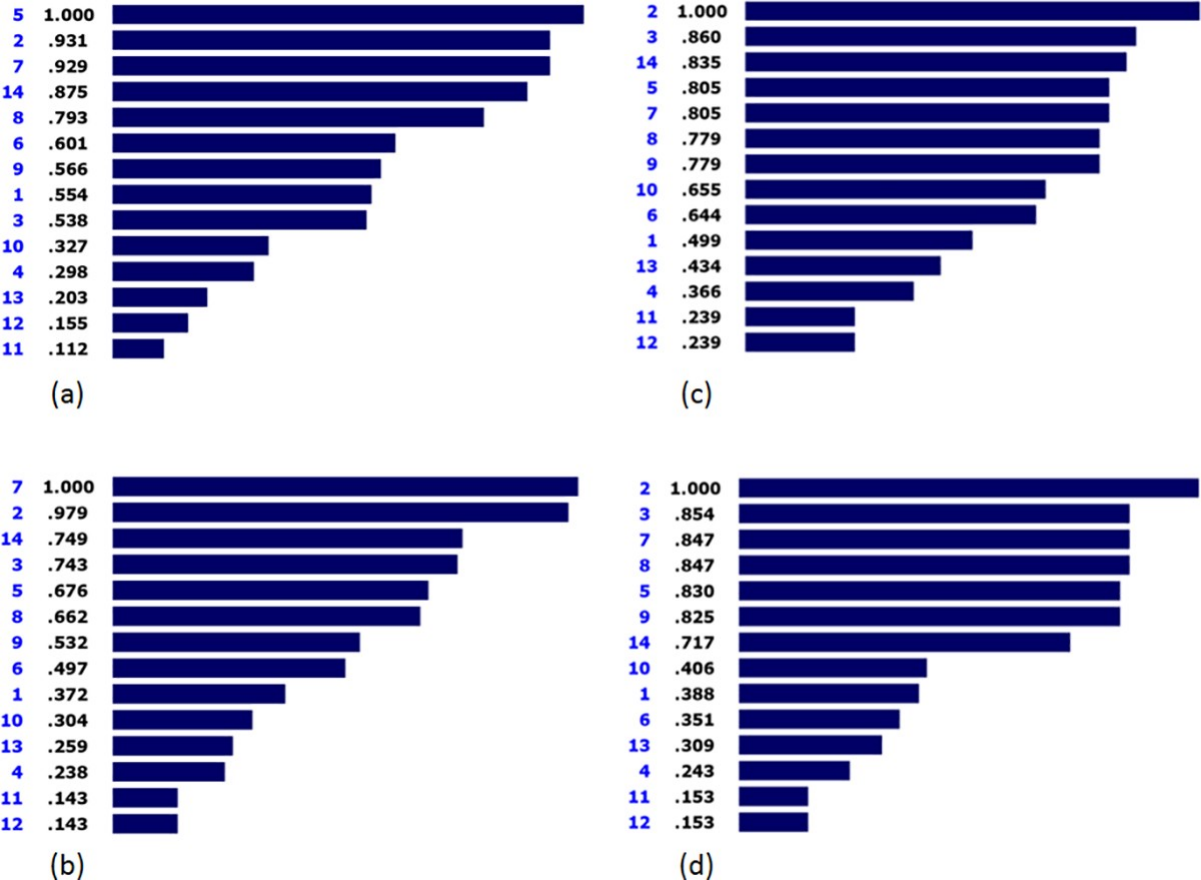
Prioritization of solutions based on the Acceptability (a), Effectiveness (b), Time (c), and Cost (d).

Fig 3A demonstrates the prioritization of solutions based on the acceptability criterion. As shown, inpatient interventions were assigned with the highest priority. Furthermore, preventive interventions (0.931), infant-specific interventions (0.929), emergency interventions (0.875), interventions until 6 years of age (0.793), and crucial prosthetics interventions (0.601) were ranked second to sixth. Additionally, sports interventions (0.203), high-tech interventions (0.155), and cosmetic prostheses (0.112) were deemed to have the lowest priority. The inconsistency index of this pairwise comparison was equal to 0.06.

### Effectiveness

Fig 3B shows the prioritized solutions considering the effectiveness criterion. As presented, infant-specific interventions (1.000), preventive interventions (0.979), and emergency interventions (0.749) had the highest priority; respectively. However, long-term interventions (0.238), cosmetic prostheses (0.143), and high-tech interventions (0.143) obtained the lowest priority. The inconsistency index of this pairwise comparison was 0.03.

### Timing

As demonstrated in Fig 3C, preventive interventions received the highest priority in accordance with the time criterion, followed by golden-period interventions (0.860), emergency interventions (0.835), inpatient interventions (0.805), and crucial prosthetics interventions (0.805). Furthermore, cosmetic prostheses (0.239) and high-tech interventions (0.239) had the lowest priority with regard to the time criterion. The inconsistency index of this pairwise comparison was also by 0.02.

### Cost

Prioritization of the retrieved solutions based on the cost criterion is presented in Fig 3D. Like the time-based prioritization, preventive interventions (1.000) and golden-period interventions (0.854) had the highest priority; respectively. Furthermore, infant-specific interventions (0.847), interventions until 6 years of age (0.847), inpatient interventions (0.830), and interventions until 18 years of age (0.825), were ranked third to sixth. Like many comparisons, cosmetic prostheses and high-tech interventions had the lowest priority with a ratio of 0.153. The inconsistency index of this pairwise comparison was 0.02.

### Feasibility

**Fig 4:**
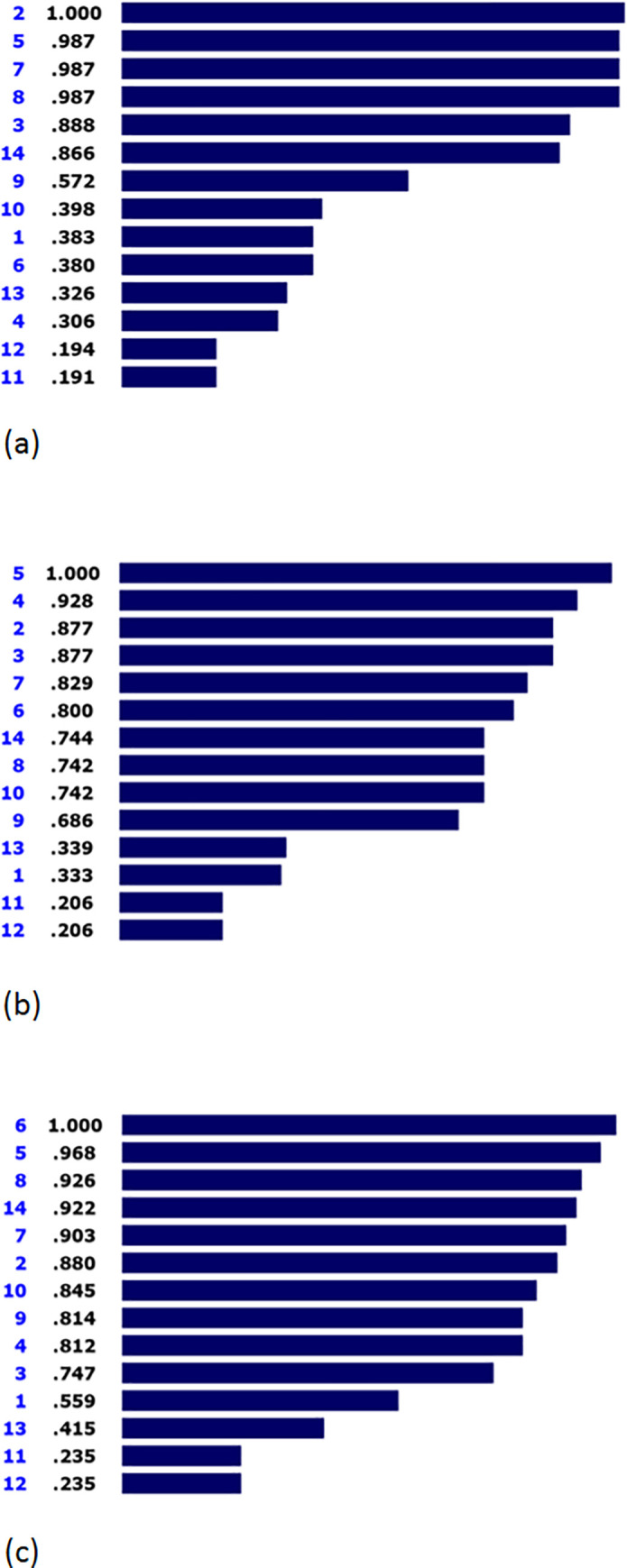
Prioritization of solutions based on Feasibility (a), Burden of disease (b), and Fairness (c).

Fig 4A displays that the preventive interventions had the highest priority based on the feasibility criterion, followed by inpatient interventions (0.987), infant-specific interventions (0.987), interventions until 6 years of age (0.987), golden-period interventions (0.888), and emergency interventions (0.866). The inconsistency index of this pairwise comparison was 0.01.

### Burden of disease

As illustrated in Fig 4B, inpatient interventions (1.000), long-term interventions (0.928), preventive interventions (0.877), and golden-period interventions (0.877) respectively obtained the highest priorities respectively for the burden of disease criterion. Infant-specific interventions (0.829), crucial prosthetic interventions (0.800), emergency interventions (0.744), and interventions until 6 years of age (0.742) were ranked fifth to eighth. The inconsistency index of this pairwise comparison was also 0.01.

### Fairness

Fig 4C presents the prioritization of solutions based on the fairness criterion. As a notable result, crucial prosthetic interventions had the highest priority regarding the fairness criterion, followed by inpatient interventions (0.968), interventions until 6 years of age (0.926), and emergency interventions (0.922). The inconsistency index of this pairwise comparison was also 0.02.

**Fig 5:**
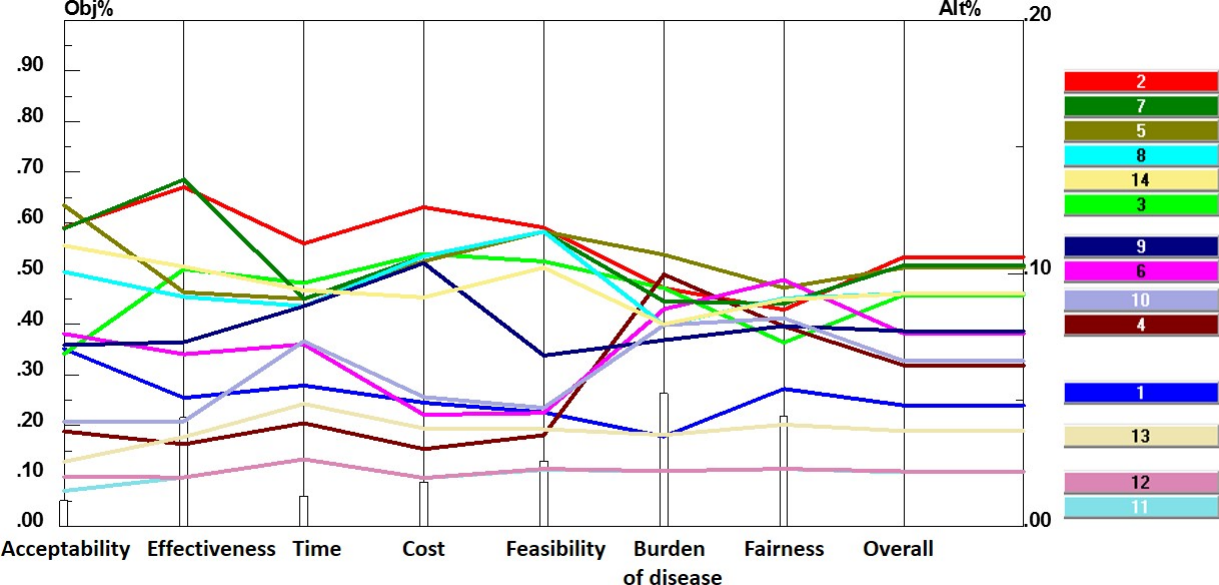
Sensitivity analysis of potential solutions based on the seven criteria (acceptability, effectiveness, time, cost, feasibility, burden of disease, and Fairness) and overall priority.

In final, Fig 5 shows the performance sensitivity analysis of the identified solutions regarding each criterion and overall priority.

## Discussion

The present study explored proper solutions to incorporate P&O services into the Iranian HBP. A number of solutions were investigated through semi-structured interviews. AHP was then employed to prioritize the proposed solutions in accordance with seven accepted criteria; including acceptability, effectiveness, time, cost, feasibility, burden of disease, and fairness. The study findings demonstrated that in overall, preventive interventions, infant-specific interventions, inpatient interventions, and interventions until 6 years of age, and emergency interventions had the highest priorities. High-tech interventions and cosmetic prostheses gained the lowest priority to be included in the HBP in Iran.

Evidence has demonstrated the preventative effects of certain orthoses, such as some foot and spine orthoses [30–32]. Although the prevailing view of policy-makers recognize these services as tertiary-level interventions [13], they can prevent many secondary aftereffects. For instance, providing orthoses to individuals with adolescent idiopathic scoliosis during their growth period can slow down its progression significantly [33]. Infant-specific interventions comprised another group of P&O services identified as a priority in this study. Orthotic management has been considered the gold standard for treatment of developmental dysplasia of the hip (DDH) in children under 6 months of age [34]. Related literature has also reported that orthotic management is an effective conservative strategy in infants with deformational plagiocephaly, and can prevent facial asymmetry and psychological disorders in coming years [35]. Early orthotic management in childhood in some conditions like clubfoot and Blount’s disease is a prerequisite for optimal outcomes and lowers the need for invasive treatment. Therefore, considering these services with preventive effects in the HBP can both reduce the burden of disease and improve equity. This process can be seen in a country like the Netherlands, trying to balance equity and efficiency as criteria for placing health care services into its basic health package [36].

It is of note that prostheses and orthoses are often required following man-made and natural disasters such as internal conflicts, traffic accidents, floods, and earthquakes [37]. Since the appropriate prostheses and orthoses services must be provided to the victims, particularly those of vulnerable groups, incorporating such services into the HBP can facilitate their utilization in line with the UHC and the SDGs. Furthermore, P&O services in the emergency phase including off-the-shelf devices to stabilize the fractures are one other category, which must be considered to prevent further impairments. The study findings revealed the importance of coverage of emergency P&O services by the HBP. Notably, the sensitivity analysis demonstrated that the emergency services were among the highest solutions based on acceptability, feasibility, and fairness criteria, indicating the smoothness of the process of inclusion of these services into the HBP.

One of the interesting findings of this study was giving priority to golden-period interventions, defined as services delivered within a certain period following a disease or injury to achieve optimal outcomes, e.g. six months after a stroke [38]. These interventions also obtained the second priority regarding time and cost criteria. Due to the limited financial resources of the Iranian health care sector [39], the coverage of such services could reduce long-term disabilities in addition to having positive clinical effects [40]. Coverage of P&O services until the age of 18 years was another proposed solution raised during interviews. Since many health care services including P&O interventions, up to the age of 18 years, can be more effective, some countries like Finland and the Netherlands have incorporated health care interventions for children under the age of 18 years into their HBPs [41, 42]. Therefore, this can be an attractive solution for Iranian health decision- and policy-makers in designing the HBP.

Developing appropriate health insurance coverage for prostheses has always been one of the common demands of amputees globally [43]. Since the costs of prostheses can be high, financial burdens are considerable [44]. Although crucial prosthetics services got the eighth priority, they were determined as the highest priority based on the fairness criterion. Given the need for prostheses by amputees to return to social life and with respect to its importance in promoting economic productivity [6], including these services into the HBP can be a firm step towards curbing burdens and increasing human dignity and equity. Therefore, incorporating the crucial prosthetics services (such as prostheses for major lower limb amputations) into the HBP can be a rational policy to promote accessibility and financial protection. Despite this, cosmetic prostheses were proposed solution that obtained the lowest priority.

Major proportions of P&O services are also used to reduce disability and to boost autonomy in the elderly. For instance, foot orthoses can have positive effects on mitigating disability and pain and improving balance in older women [45]. Furthermore, a study had reported the significant effects of spinal orthoses on enhancing balance in elderly populations [31]. Therefore, assigning P&O interventions required by the elderly in the HBP may be a proper response to aging in Iran. However, in order to reduce financial burdens on basic health insurances, long-term care insurance can be used, such as those developed in Japan [46].

Cost-effective interventions in the HBP were also one of the solutions explored in this study. Although recent investigations have reported on the cost-effectiveness of a number of P&O interventions [47, 48], some evidence has criticized mere focus on economic dimensions, arguing that social values and equity should be considered in the insurance coverage [36, 49]. In this line, cost-effective interventions achieved one of the lowest priorities based on the fairness criterion in the AHP in this study.

## Limitations and strengths

The present study has a number of limitations; we could only conduct face-to-face interview in three cities. However, the research team tried to minimize the limitations by conducting telephone interviews as well as continuous follow-up to select participants. The study would have benefitted from inputs from other health policy-makers, who unfortunately were not interested in participating in the study. The strength of this study is that it is the first one conducted on prioritization of P&O services for incorporation into HBP field. Further, applying the AHP approach was another strength of this study.

## Conclusions

In current study, a number of policy solutions for P&O services were explored and prioritized for consideration in the Iranian HBP. Our findings provide a favorable framework for other related decision- and policy-makers in Iran and other countries aiming to curb the financial burdens of service users, especially vulnerable groups.

## Supporting information

S1 Table Characteristics of participants. (DOCX)
