# Supplementary material for: Prioritizing solutions to incorporate Prosthetics and Orthotics services into Iranian health benefits package: Using an analytic hierarchy process
Source: PLoS One. 2021 Jun 8;16(6):e0253001. doi: 10.1371/journal.pone.0253001 (PMC8186777; doi:10.1371/journal.pone.0253001)
Supplement: S1 Table — (DOCX) [file pone.0253001.s001.docx]

**S1 Table.** Characteristics of participants

| Pseudonym | sex | Age  (years) | Clinical experience (years) | Education experience (years) | Executive experience (years) | Specialty | Interview format |
| --- | --- | --- | --- | --- | --- | --- | --- |
| **C001** | F | 29 | 6 | - | - | O/P | Face-to-face |
| **C002** | M | 30 | 7 | - | - | O | Face-to-face |
| **C003** | M | 38 | 14 | - | - | O/P | Telephone |
| **C004** | F | 33 | 12 | - | - | O | Telephone |
| **C005** | F | 36 | 13 | - | - | P | Telephone |
| **C006** | M | 29 | 5 | - | - | O | Face-to-face |
| **C007** | F | 36 | 12 | - | - | O/P | Telephone |
| **C008** | M | 34 | 14 | - | - | O | Face-to-face |
| **C009** | M | 42 | 20 | - | - | O/P | Skype |
| **C010** | M | 32 | 9 | - | - | O | Telephone |
| **C011** | F | 30 | 7 | - | - | O | Face-to-face |
| **C012** | F | 28 | 5 | - | - | O | Face-to-face |
| **C013** | M | 41 | 18 | - | - | O/P | Telephone |
| **F001** | M | 49 | 13 | 14 | - | O | Face-to-face |
| **F002** | F | 47 | 11 | 12 | - | O | Face-to-face |
| **F003** | M | 54 | 19 | 23 | - | P | Face-to-face |
| **F004** | M | 41 | 8 | 15 | - | 0/P | Telephone |
| **F005** | F | 43 | 10 | 13 | - | O | Face-to-face |
| **F006** | M | 45 | 14 | 16 | - | O | Telephone |
| **PM01** | M | 49 | - | - | 27 | Health Policy | Face-to-face |
| **PM02** | M | 49 | - | - | 20 | Health Policy | Telephone |
| **PM03** | F | 33 | - | - | 9 | Health Policy | Telephone |
| **PM04** | M | 51 | - | - | 28 | Health Policy | Face-to-face |
| **PM05** | M | 48 | 23 | - | 11 | Rehabilitation | Telephone |
| **PM06** | M | 49 | - | - | 24 | Health Policy | Face-to-face |
| **PM07** | F | 37 | - | - | 8 | Health Policy | Face-to-face |
| **PM08** | F | 34 | - | - | 10 | Rehabilitation | Face-to-face |
